# Gastrocnemius medialis neuromechanics during cycling at various exercise intensities

**DOI:** 10.1038/s41598-026-51412-2

**Published:** 2026-05-02

**Authors:** Josh Walker, Neil J. Cronin, Barney Wainwright, Brian Hanley, Nils Jongerius, Athanassios Bissas

**Affiliations:** 1 Carnegie School of Sport, Leeds Beckett University, Fairfax Hall 112, Headingley Campus, Leeds, LS6 3QT UK; 2 Neuromuscular Research Centre, Faculty of Sport and Health Sciences, University of Jyväskylä, Jyväskylä, Finland; 3 School of Education, Health and Sciences, University of Gloucestershire, Gloucester, UK; 4 Faculty of Health, Sport and Physical Activity, Amsterdam University of Applied Sciences, Amsterdam, The Netherlands

**Keywords:** Muscle-tendon function, Neuromuscular, Ultrasound, Loading, Triceps surae, Anatomy, Neuroscience, Physiology

## Abstract

The aim of this study was to examine triceps surae neuromechanical function during cycling at a range of exercise intensities in endurance populations with different loading regimes, and to quantify differences in morphological properties. Kinematic and neuromuscular data were collected from 29 participants (10 cyclists, nine triathletes, and 10 controls) during cycling at four intensities (150, 200, 250, 300 W). Resting muscle and tendon morphology was assessed with ultrasound. During cycling, there were no differences between groups, despite triathletes possessing thicker Achilles’ tendons. At higher intensities, ankle dorsiflexion increased (*p* < 0.001), leading to higher gastrocnemius medialis muscle-tendon unit range (*p* < 0.001), but no change in fascicle range or shortening velocity (*p* ≥ 0.919). Therefore, although there is evidence of some stretch-shortening cycle-like mechanism in the triceps surae during cycling, this does not happen at the muscle level, suggesting that energy storage and return could occur predominantly in the non-contractile series-elastic elements. Despite this, there were no differences detected between trained and untrained individuals in gastrocnemius medialis neuromechanical behavior at the exercise intensities tested, even though triathletes possessed a higher Achilles’ tendon thickness.

**Supplementary Information:**

The online version contains supplementary material available at 10.1038/s41598-026-51412-2.

## Introduction

The study of muscle-tendon unit (MTU) neuromechanical behavior during human locomotion has received attention in the last two decades, especially in more recent years because of advancements in the data collection and processing capabilities of systems measuring dynamic muscle architecture in vivo. Quantifying muscle architecture during locomotion has enabled researchers to understand the interaction between contractile (CE) and series-elastic elements (SEE) of MTUs in human gait^1–4^ or in jumping/hopping movements^5–7^. It is generally accepted that muscle actions with higher fascicle lengthening and shortening velocities induce a higher metabolic cost than actions with lower velocities^8–10^. Therefore, from the perspective of energetic efficiency, a pattern of MTU interaction that minimizes active fascicle lengthening and shortening, for a given MTU lengthening and shortening, would be advantageous. This appears to be the case in human lower limbs, as shown experimentally in the gastrocnemius medialis (GM) MTU during gait^3^ and in jumping tasks^5^.

MTU behavior is affected by its morphological and mechanical properties (e.g., resting muscle architecture, tendon thickness, passive properties), which differ between trained and untrained individuals^11–14^, and even between groups of endurance-trained populations who have been exposed to different long-term loading regimes^15,16^. In addition to changes in resting properties, chronic adaptations to specific loading strategies through endurance training are evident in active MTU function. Seminal work by Herzog, et al. ^17^ showed that length-tension properties of rectus femoris are different in runners compared with cyclists/speed skaters, where the differences were attributed to the MTU length that the rectus femoris operates at, indicating a sport- or movement-specific adaptation to meet the demands of the activity. This specific adaptation likely leads to a refined or optimized MTU interaction for performance in that activity, which might be problematic in populations who perform multiple forms of endurance exercise, such as triathletes. Trained triathletes have a higher ankle joint stiffness (governed largely by the triceps surae MTU) during slow, passive dorsiflexion movements when compared with trained cyclists and untrained controls^16^. Whilst this difference could be attributed to the relatively higher Achilles’ tendon (AT) loading magnitudes during running than cycling^18,19^, or to the plantarflexed position of the ankle joint during swimming leading to increased AT stiffness, its impact on triceps surae MTU function during cycling specifically remains unclear. It is plausible that the higher stiffness of the triceps surae MTUs could modify the interaction between CE and SEE during submaximal cycling exercise, thus possibly altering energetic efficiency and compromising performance.

Triceps surae MTU length changes are affected by both ankle and knee joint kinematics^20^. Previous research has shown that the ankle joint shifts into a more dorsiflexed position as cycling intensity increases^21,22^. However, the mechanism underpinning this response remains unclear. Given that triceps surae MTUs typically work on the ascending limb of the length-tension relationship^23,24^, perhaps this increased dorsiflexion is a strategy to increase fascicle length (*L*_*fasc*_) of muscles such as GM, thus increasing their force potential. Alternatively, it could also be speculated that the ankle joint could be “forced” into dorsiflexion at higher cycling intensities, as opposed to this change being a voluntary response towards optimization. During the downstroke during cycling, the ankle experiences plantarflexor joint moments because the point of force application (the pedal-shoe interface, which is generally a near-fixed point of force application) is anterior to the ankle joint center^25^; given this moment arm does not change, and pedal forces increase at higher cycling intensities^26^, higher triceps surae strength and/or stiffness would be required to maintain ankle position. Given certain cycling populations (triathletes) possess higher passive ankle stiffness, it could be suggested that ankle angle would respond to a lesser extent in this group if the latter suggestion were true. However, direct comparisons between trained populations across cycling intensities have not been explored.

Biarticular muscles, particularly the plantarflexors (GM and gastrocnemius lateralis, GL), are responsible for the transfer of forces from proximal to distal joints, as well as altering the direction of force application^27^. This is particularly evident in a constrained movement such as cycling, where the orientation of forces about the single point of force application heavily influences power output. Therefore, the structure and function of the MTUs responsible for energy transfer and the direction of force application are particularly of interest when seeking to better understand cycling performance. Some previous work has examined muscle mechanics of GM during cycling at different intensities, showing that fascicle and MTU shortening velocities tend to increase with increasing intensity^28,29^, although it remains unclear how transferable these findings are across different cycling populations given the aforementioned differences in passive properties. Nonetheless, these studies still provide novel insights into GM MTU function, showing that the muscles spanning the ankle joint potentially have a key role in modulating increases in exercise intensity by altering their mechanical behavior (e.g., gearing). Besides these studies, there is notably less research on triceps surae MTU function during cycling despite its importance for energy transfer and the orientation of pedal force^30^, particularly concerning between-group comparisons. Instead, research has tended to focus more on the monoarticular vastus lateralis (VL) MTU^31–33^ because of its role in force generation. The aim of the current study was to examine muscle-tendon neuromechanical behavior of the triceps surae during cycling in trained cyclists (CYC), triathletes (TRI), and untrained individuals (CON) across a range of isoinertial cycling intensities (150, 200, 250, and 300 W) under ecologically valid conditions (e.g., self-selected cadences).

## Results

### Muscle-tendon function during cycling

Two participants from CON were omitted from this part of the analysis as they were unable to maintain the power output in all four conditions. For all included participants, cadence was 87 ± 5 revolutions per min (rpm), 88 ± 5 rpm, 88 ± 6 rpm, and 89 ± 6 rpm when cycling at 150, 200, 250, and 300 W, respectively. There was no statistically significant main effect of group on cadence (*F*_*2,24*_ = 2.54, *p* = 0.100, *η*_*p*_^*2*^ *=* 0.175 [large]), although there was a main effect of condition (*F*_*3,23*_ = 3.86, *p* = 0.023, *η*_*p*_^*2*^ *=* 0.138 [medium]). However, post-hoc testing showed no individual differences between conditions (*p* ≥ 0.05) using the current sample. There was no group × condition interaction for cadence (*p* = 0.413, *η*_*p*_^*2*^ *=* 0.078 [medium]).

**Fig. 1:**
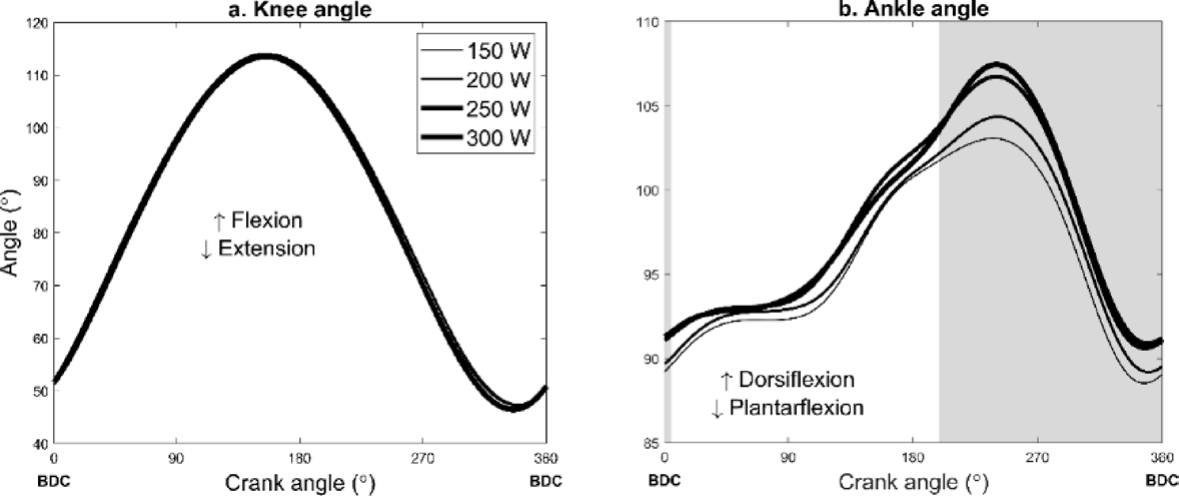
Joint angle profiles (a. knee angle – left subplot, and b. ankle angle – right subplot), presented as a function of crank angle, for all participants at the four exercise intensities. Higher intensities are denoted by thicker lines. For clarity, groups have been pooled together to show main effects of exercise intensity (*n* = 26, one participant removed because of erroneous knee angle data). Larger values depict greater angles of flexion (i.e., knee flexion, and more ankle dorsiflexion). Gray shaded areas indicate locations of suprathreshold clusters determined from SPM analyses, where a main effect of condition was found (*p* < 0.05). BDC = bottom-dead-center (i.e., the bottom of the pedal stroke, when the pedal is directly below the bottom bracket of the ergometer). Statistical results of SPM analyses can be found in the Supplementary Material (Supplemental Fig. S1).

There was a main effect of condition on ankle angle curves from 199-6° of the crank cycle (*p* < 0.001), with the ankle joint adopting a more dorsiflexed position, particularly during the downstroke (Fig. 1). There was no main effect of group, or any significant group × condition interactions, for joint angle-crank angle curves (Fig. 1; Supplemental Fig. S1).

**Fig. 2:**
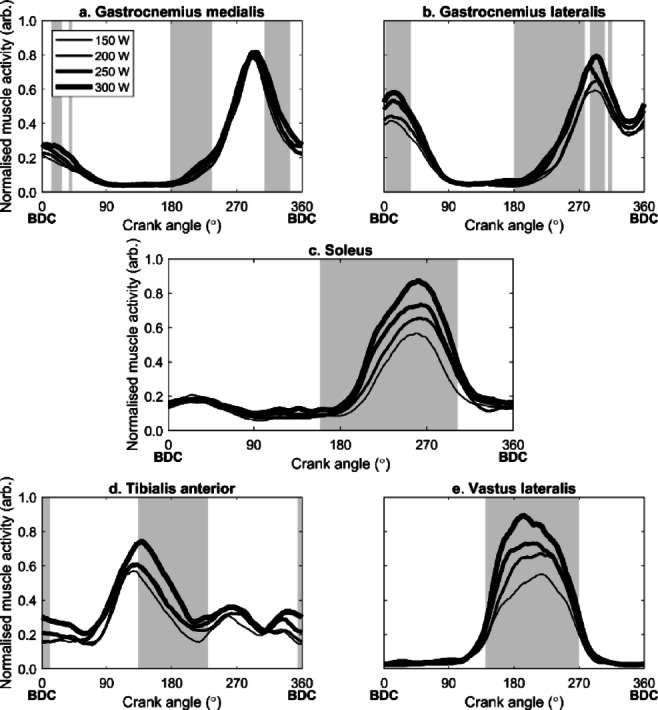
Muscle activity profiles (a. gastrocnemius medialis – top-left subplot, b. gastrocnemius lateralis – top-right subplot, c. soleus – middle subplot, d. tibialis anterior – bottom-left subplot, and e. vastus lateralis – bottom-right subplot), presented as a function of crank angle, for all participants at the four exercise intensities. Higher intensities are denoted by thicker lines. For clarity, groups have been pooled together to show main effects of exercise intensity (*n* = 27 for gastrocnemius medialis, gastrocnemius lateralis, and tibialis anterior, *n* = 26 for vastus lateralis, and *n* = 25 for soleus, participants removed because of excessively noisy signals). To allow comparison between conditions, data have been normalized to the maximal individual activity detected during the 300 W condition, so are presented as arbitrary units (arb.). Gray shaded areas indicate locations of suprathreshold clusters determined from SPM analyses, where a main effect of condition was found (*p* < 0.05). BDC = bottom-dead-center (i.e., the bottom of the pedal stroke, when the pedal is directly below the bottom bracket of the ergometer). Statistical results of SPM analyses can be found in the Supplementary Material (Supplemental Fig. S2).

There was a main effect of condition on the normalized activity of all five muscles measured (Table 1; η_*p*_^*2*^ *≥* 0.332 [all large]). Post-hoc testing showed that for GM, normalized activity was higher at 300 W than all other conditions (*p* ≤ 0.003) and was also higher at 250 W compared to 150 W (*p* = 0.017). Similar findings were observed for GL and soleus (SOL), with normalized activity generally increasing with exercise intensity (Table 1). Regarding TA, post-hoc tests showed higher activity at 300 W compared to all other conditions (*p* ≤ 0.002), whilst for VL, all conditions were different from each other (*p* ≤ 0.004). Additionally, there was a main effect of group for VL activity, although post-hoc testing did not detect any individual differences. There were no group × condition interactions for normalized muscle activity (Table 1). In the time-series analyses, a main effect of condition was found for GM mainly after top-dead-center and in the downstroke, from 178–236° and 308–344° (*p* < 0.001), with shorter main effects during the upstroke around 11–29° (*p* = 0.014) and 38–43° (*p* = 0.043) of the crank cycle (Fig. 2). Main effects of condition were found for GL from 3–38°, 180–278°, 285–306°, and 310–316° (*p* ≤ 0.040; Fig. 2). For SOL, a single suprathreshold cluster was found from 159–302° (*p* < 0.001). TA showed a main effect of condition predominantly around top-dead-center (134–200°, *p* < 0.001), with a shorter suprathreshold cluster around BDC (354 − 12°, *p* ≤ 0.043; Fig. 2). VL showed a single suprathreshold cluster around 141–270° (*p* < 0.001; Fig. 2). There was no main effect of group for any muscle’s activity-crank angle curve, with no group × condition interactions (Supplemental Fig. S2).

**Table 1:** Muscle activity data for each group at the different cycling intensities.

		CYC	TRI	CON	ANOVA	Post-hoc
GM activity (arb.)	150 W 200 W 250 W 300 W	0.21 ± 0.05 0.23 ± 0.05 0.23 ± 0.05 0.25 ± 0.05	0.22 ± 0.07 0.22 ± 0.05 0.23 ± 0.06 0.26 ± 0.07	0.21 ± 0.07 0.23 ± 0.06 0.23 ± 0.06 0.24 ± 0.08	Group: *F*_*2,24*_ = 0.03, *p* = 0.967, *η*_*p*_^*2*^ *=* 0.003 **Condition**: ***F***_***3,23***_ **= 14.83**, ***p*** **< 0.001**, ***η***_***p***_^***2***^ ***=*** **0.382** Interaction: *p* = 0.684, *η*_*p*_^*2*^ *=* 0.052	150 W < 250, 300 W; 200 W < 300 W; 250 W < 300 W
GL activity (arb.)	150 W 200 W 250 W 300 W	0.24 ± 0.08 0.23 ± 0.07 0.30 ± 0.07 0.31 ± 0.06	0.24 ± 0.07 0.26 ± 0.06 0.27 ± 0.07 0.31 ± 0.07	0.23 ± 0.07 0.26 ± 0.09 0.29 ± 0.09 0.33 ± 0.07	Group: *F*_*2,24*_ = 0.05, *p* = 0.949, *η*_*p*_^*2*^ *=* 0.004 **Condition (GG)**: ***F***_***3,23***_ **= 17.82**, ***p*** **< 0.001**, ***η***_***p***_^***2***^ ***=*** **0.426** Interaction: *p* = 0.365, *η*_*p*_^*2*^ *=* 0.084	150 W < 250, 300 W; 200 W < 300 W; 250 W < 300 W
SOL activity (arb.)	150 W 200 W 250 W 300 W	0.21 ± 0.07 0.22 ± 0.10 0.24 ± 0.06 0.29 ± 0.05	0.18 ± 0.05 0.21 ± 0.04 0.24 ± 0.05 0.30 ± 0.05	0.19 ± 0.07 0.24 ± 0.07 0.27 ± 0.10 0.28 ± 0.05	Group: *F*_*2,22*_ = 0.14, *p* = 0.869, *η*_*p*_^*2*^ *=* 0.013 **Condition (GG)**: ***F***_***3,21***_ **= 28.06**, ***p*** **< 0.001**, ***η***_***p***_^***2***^ ***=*** **0.561** Interaction: *p* = 0.322, *η*_*p*_^*2*^ *=* 0.099	150 W < all other conditions; 200 W < 300 W; 250 W < 300 W
TA activity (arb.)	150 W 200 W 250 W 300 W	0.33 ± 0.19 0.31 ± 0.15 0.33 ± 0.10 0.37 ± 0.07	0.25 ± 0.04 0.29 ± 0.07 0.34 ± 0.11 0.40 ± 0.10	0.19 ± 0.07 0.26 ± 0.10 0.26 ± 0.09 0.32 ± 0.11	Group: *F*_*2,24*_ = 1.87, *p* = 0.176, *η*_*p*_^*2*^ *=* 0.135 **Condition (GG)**: ***F***_***3,23***_ **= 11.91**, ***p*** **< 0.001**, ***η***_***p***_^***2***^ ***=*** **0.332** Interaction: *p* = 0.263, *η*_*p*_^*2*^ *=* 0.101	All other conditions < 300 W
VL activity (arb.)	150 W 200 W 250 W 300 W	0.20 ± 0.03 0.25 ± 0.07 0.26 ± 0.03 0.30 ± 0.03	0.16 ± 0.03 0.19 ± 0.03 0.24 ± 0.03 0.27 ± 0.03	0.16 ± 0.04 0.20 ± 0.07 0.23 ± 0.05 0.28 ± 0.03	**Group**: ***F***_***2,23***_ **= 4.02**, ***p*** **= 0.032**, ***η***_***p***_^***2***^ ***=*** **0.259** **Condition (GG)**: ***F***_***3,22***_ **= 88.97**, ***p*** **< 0.001**, ***η***_***p***_^***2***^ ***=*** **0.795** Interaction: *p* = 0.483, *η*_*p*_^*2*^ *=* 0.074	All conditions different to other conditions; No between-group differences

Values are means ± SD. CYC = cyclists; TRI = triathletes; CON = controls; GG = Greenhouse-Geisser correction applied; GM = gastrocnemius medialis; GL = gastrocnemius lateralis; SOL = soleus; TA = tibialis anterior; VL = vastus lateralis. Bonferroni correction applied to post-hoc testing. Data are normalized to the peak activity detected during the 300 W condition for each individual muscle, hence data are presented in arbitrary units (arb.).

**Fig. 3:**
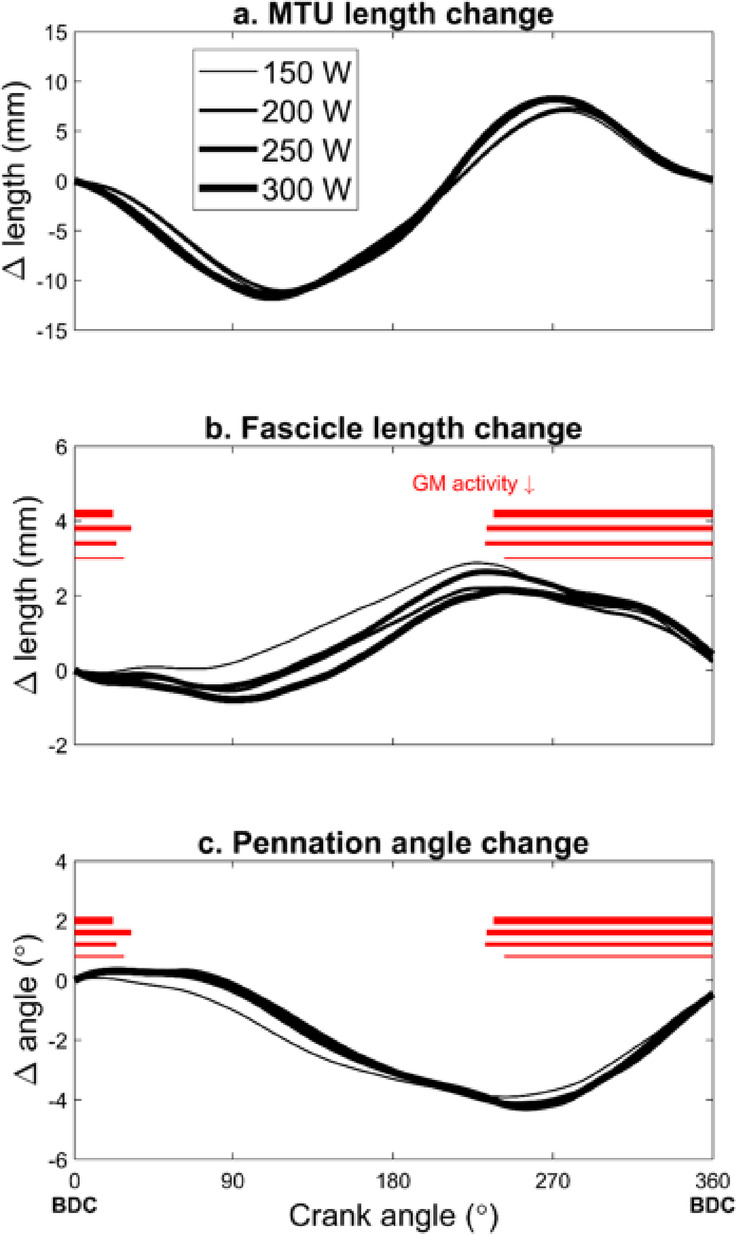
Muscle-tendon unit mechanical characteristics (a. MTU length change – top subplot, b. fascicle length change – middle subplot, and c. pennation angle change – bottom subplot), presented as a function of crank angle, for all participants at the four exercise intensities. Higher intensities are denoted by thicker lines. For clarity, groups have been pooled together to show main effects of exercise intensity (*n* = 26, one participant removed because of erroneous knee angle data). Data are displayed relative to bottom-dead-center. Horizontal red bars on the middle and bottom subplots indicate the location of the main activity burst of the gastrocnemius medialis, thus indicating fascicle mechanical behavior whilst the muscle is active. BDC = bottom-dead-center (i.e., the bottom of the pedal stroke, when the pedal is directly below the bottom bracket of the ergometer). Statistical results of SPM analyses can be found in the Supplementary Material (Supplemental Fig. S3).

There was a significant main effect of condition on MTU range (*η*_*p*_^*2*^ = 0.368 [large]), with post-hoc tests showing the 150 W condition to induce a lower MTU range than all other conditions (*p* ≤ 0.038; Table 2), whilst 200 W was also lower than 300 W (*p* < 0.001). Despite the main effect of condition on MTU range, there was no main effect found for fascicle range or pennation angle range (Table 2; *η*_*p*_^*2*^ ≤ 0.026 [small-to-negligible]). There were also no significant main effects of group on any MTU mechanical variable; however, there was an interaction effect for fascicle range and mean fascicle shortening velocity (*p* ≤ 0.044, *η*_*p*_^*2*^ *=* 0.161 [large]; Table 2), although no significant differences were found in post-hoc testing using the current sample. According to SPM analyses, there were no main effects of group or condition throughout the crank cycle for MTU length change, fascicle length change, or pennation angle change, with no significant group × condition interaction effects (Supplemental Fig. S3). MTU length change follows a similar waveform during the four conditions, and there is a relatively consistent pattern of fascicle mechanical behavior (Fig. 3). Figure 3 also shows the portions of the crank cycle where the main GM activity burst was situated, showing that activity occurred after the GM fascicle’s lengthening phase. Following the activity onset, *L*_*fasc*_ decreased and pennation angle increased.

**Table 2:** Muscle-tendon unit mechanical data for each group at the different cycling intensities.

		CYC	TRI	CON	ANOVA	Post-hoc
MTU range (mm)	150 W 200 W 250 W 300 W	21.20 ± 5.20 21.11 ± 4.90 22.21 ± 4.81 22.99 ± 6.38	18.80 ± 6.12 23.01 ± 9.08 20.54 ± 8.70 24.79 ± 8.61	19.91 ± 7.33 22.51 ± 6.69 24.32 ± 6.18 26.38 ± 6.21	Group: *F*_*2,23*_ = 0.15, *p* = 0.865, *η*_*p*_^*2*^ *=* 0.013 **Condition**: ***F***_***3,22***_ **= 13.40**, ***p*** **< 0.001**, ***η***_***p***_^***2***^ ***=*** **0.368** Interaction: *p* = 0.054, *η*_*p*_^*2*^ *=* 0.177	150 W < all other conditions; 200 W < 300 W
Fascicle range (mm)	150 W 200 W 250 W 300 W	5.89 ± 3.45 5.01 ± 3.10 4.95 ± 3.45 4.00 ± 2.52	5.01 ± 2.84 5.51 ± 4.30 5.48 ± 4.09 7.13 ± 5.11	4.01 ± 2.79 4.21 ± 3.06 4.67 ± 3.80 3.71 ± 3.03	Group: *F*_*2,24*_ = 0.52, *p* = 0.600, *η*_*p*_^*2*^ *=* 0.042 Condition: *F*_*3,23*_ = 0.64, *p* = 0.991, *η*_*p*_^*2*^ *=* 0.001 **Interaction**: ***p*** **= 0.005**, ***η***_***p***_^***2***^ ***=*** **0.224**	No significant differences
Pennation angle range (°)	150 W 200 W 250 W 300 W	4.89 ± 3.10 4.14 ± 1.87 4.24 ± 2.30 4.03 ± 2.28	6.19 ± 5.61 6.98 ± 5.50 6.42 ± 6.60 6.91 ± 5.48	4.19 ± 2.01 5.71 ± 1.87 6.89 ± 6.58 6.73 ± 7.44	Group: *F*_*2,24*_ = 0.73, *p* = 0.491, *η*_*p*_^*2*^ *=* 0.058 Condition (GG): *F*_*3,23*_ = 0.64, *p* = 0.517, *η*_*p*_^*2*^ *=* 0.026 Interaction: *p* = 0.365, *η*_*p*_^*2*^ *=* 0.084	n/a
Mean fascicle shortening velocity (mm/s)	150 W 200 W 250 W 300 W	17.98 ± 14.57 15.72 ± 10.35 16.31 ± 12.73 12.87 ± 8.33	17.21 ± 11.14 16.74 ± 12.11 16.15 ± 11.05 20.69 ± 13.71	11.39 ± 7.33 12.32 ± 8.71 14.65 ± 14.22 11.71 ± 10.34	Group: *F*_*2,24*_ = 0.49, *p* = 0.616, *η*_*p*_^*2*^ *=* 0.040 Condition (GG): *F*_*3,23*_ = 0.17, *p* = 0.919, *η*_*p*_^*2*^ *=* 0.007 **Interaction**: ***p*** **= 0.044**, ***η***_***p***_^***2***^ ***=*** **0.161**	No significant differences

Values are means ± SD. CYC = cyclists; TRI = triathletes; CON = controls; GG = Greenhouse-Geisser correction applied; MTU = muscle-tendon unit. Bonferroni correction applied to post-hoc testing. Range data are presented as the maximum value minus the minimum value throughout the pedal cycle. Mean fascicle shortening velocity is presented as the mean value of the shortening portions of the fascicle velocity-crank angle curve.

## Muscle and tendon morphology

There was no significant main effect of group for AT resting length when presented in absolute terms or normalized to shank length (Table 3; *η*_*p*_^*2*^ ≤ 0.037 [small]). There was a main effect of group for AT thickness, where TRI displayed a higher thickness than CYC (*p* = 0.031). There was no main effect of group for GM or SOL muscle thickness, but there was for GL (Table 3), where TRI had a thicker GL than CYC (*p* = 0.031). There was no significant main effect of group for pennation angle or *L*_*fasc*_ at any of the measured sites (Table 3).

**Table 3:** Data for muscle and tendon morphological properties across the three groups.

	CYC	TRI	CON	ANOVA	Post-hoc
AT length (mm)	229 ± 27	228 ± 23	219 ± 22	*F*_*2,26*_ = 0.50, *p* = 0.611, *η*_*p*_^*2*^ *=* 0.037	*n*/a
AT length (% shank length)	51.3 ± 5.0	49.5 ± 3.7	50.4 ± 4.0	*F*_*2,26*_ = 0.44, *p* = 0.648, *η*_*p*_^*2*^ *=* 0.033	n/a
AT thickness (mm)	5.5 ± 0.4	6.5 ± 1.1	5.6 ± 0.6	***F***_***2,25***_ **= 4.52**, ***p*** **= 0.021**, ***η***_***p***_^***2***^ ***=*** **0.265**	TRI > CYC
GM muscle thickness (mm)	21.5 ± 3.2	23.1 ± 2.6	22.8 ± 2.7	*F*_*2,26*_ = 0.85, *p* = 0.441, *η*_*p*_^*2*^ *=* 0.061	n/a
GM pennation angle (°)	18.5 ± 2.9	17.9 ± 4.1	20.8 ± 1.7	*F*_*2,26*_ = 2.54, *p* = 0.098, *η*_*p*_^*2*^ *=* 0.163	n/a
GM *L*_*fasc*_ (mm)	70.8 ± 14.3	78.1 ± 18.5	66.4 ± 10.1	*F*_*2,26*_ = 1.55, *p* = 0.231, *η*_*p*_^*2*^ *=* 0.106	n/a
GL muscle thickness (mm)	15.6 ± 2.0	18.9 ± 3.3	16.2 ± 2.4	***F***_***2,26***_ **= 4.29**, ***p*** **= 0.024**, ***η***_***p***_^***2***^ ***=*** **0.248**	TRI > CYC
GL pennation angle (°)	11.1 ± 2.1	12.4 ± 1.8	13.5 ± 2.7	*F*_*2,26*_ = 2.89, *p* = 0.074, *η*_*p*_^*2*^ *=* 0.182	n/a
GL *L*_*fasc*_ (mm)	75.8 ± 14.3	84.0 ± 21.4	69.9 ± 9.2	*F*_*2,26*_ = 1.96, *p* = 0.161, *η*_*p*_^*2*^ *=* 0.131	n/a
SOL muscle thickness (mm)	14.4 ± 2.2	15.8 ± 3.5	14.4 ± 3.7	*F*_*2,26*_ = 0.56, *p* = 0.580, *η*_*p*_^*2*^ *=* 0.043	n/a
SOL pennation angle (°)	18.9 ± 4.5	16.7 ± 4.9	20.4 ± 3.2	*F*_*2,26*_ = 1.69, *p* = 0.205, *η*_*p*_^*2*^ *=* 0.119	n/a
SOL *L*_*fasc*_ (mm)	44.9 ± 12.6	52.5 ± 14.8	38.5 ± 8.9	*F*_*2,26*_ = 2.96, *p* = 0.070, *η*_*p*_^*2*^ *=* 0.192	n/a

Values are means ± SD. CYC = cyclists; TRI = triathletes; CON = controls; AT = Achilles’ tendon; GM = gastrocnemius medialis; GL = gastrocnemius lateralis; SOL = soleus; *L*_*fasc*_ = fascicle length. Bonferroni correction applied to post-hoc testing. To present AT lengths normalized to shank length, shank length was measured as the distance between the lateral tibial condyle and the lateral malleolus of the ankle.

## Discussion

The aim of this study was to quantify triceps surae neuromechanical behavior during cycling at four absolute exercise intensities and at self-selected cadences in cyclists, triathletes, and untrained individuals. The study also compared MTU morphological characteristics in the three groups to understand population differences in resting MTU properties. Responses to increases in exercise intensity were generally characterized by a more dorsiflexed ankle and an increase in muscle activity amplitude, but no changes in muscle architectural characteristics were found. Other than higher AT and GL thickness in TRI (compared to CYC only), no other differences were detected between groups for triceps surae morphology, and neuromechanical behavior (joint kinematics, muscle activity patterns, MTU mechanical patterns) during cycling at all absolute intensities were not found to be different using the present study design.

Even though there were no differences found between groups for kinematic or neuromechanical characteristics during cycling, there were consistently significant main effects of condition. First of all, the ankle joint shifted into a more dorsiflexed position at higher cycling intensities, particularly during the downstroke, in accordance with previous research^21,22^. This change in ankle angle is suggestive of an increase in the GM MTU operating length^25^, given knee kinematics did not change. Indeed, an increase in the GM MTU range was observed, although no suprathreshold clusters for MTU length change were detected throughout the pedal cycle. It is possible that variations in absolute knee angle, ankle angle, and shank length between participants meant that group-level MTU length changes throughout the pedal cycle were not detected. It is also plausible to suggest that presenting absolute MTU length over time would have accounted for such variations and might have been different between conditions. However, we chose to present only length change data to be consistent with the convention for *L*_*fasc*_ change data. Previous research has suggested that *L*_*fasc*_ change data are more reliable than absolute values^34^, whereas derivatives such as fascicle range and shortening velocity remain unaffected. Although beyond the scope of the current study, understanding the association between muscle (agonist-antagonist) co-contraction during cycling^35^ and fascicle mechanical behavior might have offered additional insight.

**Fig. 4:**
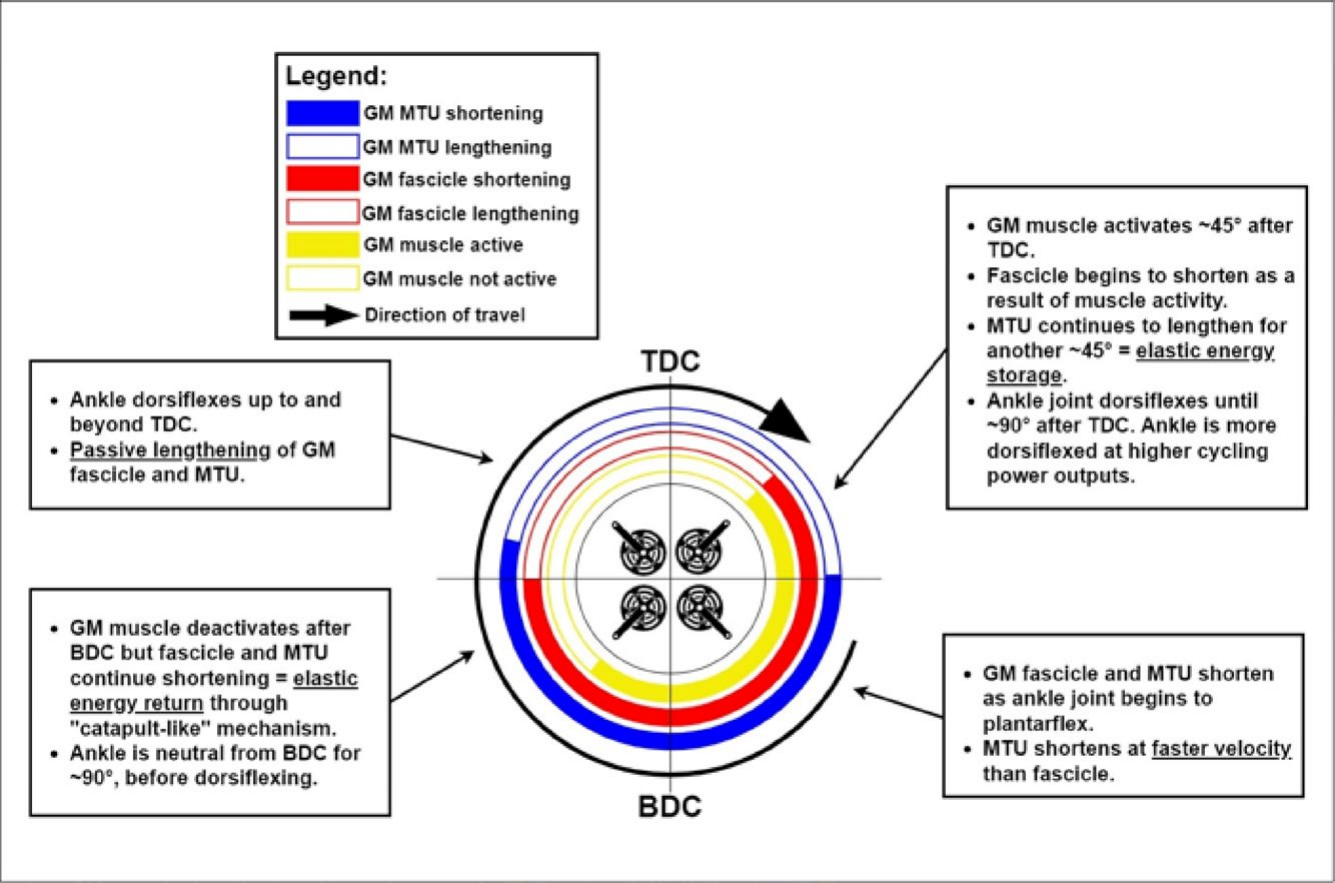
Infographic overview of GM MTU neuromechanical behavior during cycling, including GM muscle activity (yellow), GM fascicle lengthening and shortening (red), and GM MTU lengthening and shortening (blue). BDC = bottom-dead-center (i.e., the bottom of the pedal stroke, when the pedal is directly below the bottom bracket of the ergometer); TDC = top-dead-center (i.e., the top of the pedal stroke, when the pedal is directly above the bottom bracket of the ergometer).

Despite the change in MTU range across intensities, there was no such increase in fascicle range or pennation angle range found using the current design, with no significant changes in *L*_*fasc*_-crank angle or pennation angle-crank angle curves. Based on the two-component (CE and SEE) model used in the current study, an increase in MTU range without a concomitant increase in fascicle range or pennation angle change implies and increase in SEE range, providing some indirect evidence of elastic energy storage in the SEE during cycling, aligning with some previous suggestions^25^. The additional stretch and recoil of the MTU being attributed to the SEE (tendon and/or aponeuroses) and facilitated by the CE not altering length change between intensities is often referred to as the CE acting in a “strut-like” manner^36^, although this was not specifically quantified in the current study. However, this pattern of muscle-tendon interaction is in alignment with other studies of biarticular MTUs like GM^3,5,6^ in gait and jumping, showing that stretch-shortening cycles^37^ do not happen at the muscle level, but do at the MTU level. This behavior is useful for transferring energy between body segments^27^, and also means that fascicles can operate at relatively lower shortening velocities, possibly generating more force at a lower energetic cost^3^. Future research might wish to clarify this interpretation by analyzing the monoarticular SOL, which might be more likely to display a classical stretch-shortening cycle at the muscle level. It should be emphasized that in order to maintain this pattern for force transfer at higher cycling intensities, an increase in the amplitude of muscle activity was required. The timing of this activity in GM occurred after top-dead-center and was shortly followed by active fascicle shortening and an increase in pennation angle (Fig. 3). The muscle-tendon neuromechanical behavior of GM during cycling is depicted in Fig. 4 and is characterized as follows: MTU lengthening is much larger than fascicle lengthening; during the downstroke, the fascicle actively shortens while the MTU continues to lengthen; towards BDC, there is a recoil of both fascicle and MTU.

The comparison between trained and untrained populations at self-selected cadences revealed no statistically significant differences for MTU and fascicle range, dynamic muscle architectural changes (e.g., pennation angle), and fascicle shortening velocities when comparing between groups (small effect sizes). Given that MTU length changes are computed from joint angle data^20^, the non-significant differences in joint kinematics between groups could largely explain this. However, differences in fascicle mechanical characteristics were perhaps expected, given that previous research has shown that the triceps surae MTU behaves differently under passive conditions between these populations^16^. Additionally, lower fascicle shortening velocities, or less muscle activity for a given shortening velocity, might have been expected in a more trained population^38^, but this was not found using the current study design (including the approach to EMG signal processing, which was perhaps better suited to comparing exercise intensities than between-group differences). Given the study used absolute, as opposed to relative, cycling intensities, it is possible that the trained populations were exercising at a lower relative intensities than the untrained groups, implying that there might have been a lesser requirement to preserve energy in CYC and TRI, although further research using absolute and relative exercise intensities is warranted to explore this concept further.

The higher AT thickness and GL muscle thickness in TRI possibly indicates a loading response to the types of training they are exposed to, namely running, which has higher AT loading magnitudes compared with cycling^18,19^, thus providing a stimulus possibly sufficient to cause morphological change (although directly implying cause-and-effect is beyond the scope of this study). AT morphological responses (AT cross-sectional area) have been demonstrated in trained runners^12^, and our findings might corroborate those. Additionally, the thicker AT in TRI is possibly related to the higher passive stiffness reported previously^16^, as a tendon’s size (i.e., thickness or cross-sectional area) directly affects its stress-strain properties. This might be supported by a review article^39^ that suggests loading magnitude is a key component of tendon mechanical, material, or morphological adaptation, and the findings presented here possibly suggest that triathlon training (either the specific types of loading, such as running, or the combination of different exercises that make up training volume) meet this loading magnitude. However, it remains unclear whether these morphological differences lead to changes in neuromechanical behavior during cycling.

This study was not without its limitations. First, it should be acknowledged that, as with many in-depth neuromechanical studies in trained populations, modest group sizes may limit sensitivity to detect small between-group or interaction effects. However, despite this limitation and various other confounding factors, the repeated-measures design and the consistency of findings across multiple independent outcome measures provide some suggestion that any undetected effects could be small. It was also a cross-sectional study, which means that any observed differences between groups were not necessarily chronic adaptations but might have been present in the participants before they commenced training and competing in their respective events. Nonetheless, these retrospective analyses are necessary to provide unique information about specialist populations without the time-consuming nature of longitudinal studies (some participants had over 10 years of training exposure). It should also be noted that the self-reported training volumes in TRI and CON were not significantly different for either cycling or running, but there were notable differences in the average age of each group (between CON and trained groups). This could mean that some homogeneity (in training volume) between these groups, and the effects of age on key mechanical properties like tendon stiffness^40^, might have masked some of the true effects of chronic exposure. However, the reported competitive history still might have altered MTU morphology in TRI only, so we feel the group-level distinction, regardless of age or training volume discrepancies, remains somewhat valid. There was also notable within-group variability for certain metrics reported in this study, suggesting some heterogeneity within groups (either in performance or in MTU strategy) which might have further masked between-group comparisons. Further research should expand on this by exploring individual MTU strategies across cycling intensities, including MTU mechanical behavior and muscle (co-)activity patterns, regardless of training history. Additionally, the isoinertial cycle ergometer used in the current study meant that within- and between-revolution variation in pedal speed was more likely to occur than in studies with isokinetic ergometers (although there were no significant cadence differences found between groups or conditions, there were large and medium effect sizes, respectively). Pedal velocity is relatively constant across a pedal cycle when using circular chainrings^41^, and isoinertial ergometers maintain better ecological validity than isokinetic ergometers. This study was the first to show neuromechanical changes at freely chosen cadences during isoinertial cycling. Despite this, the large and medium effect sizes for between-group and between-condition comparisons, respectively, pose a notable limitation to the study given that cadence will likely affect the MTU (and possibly also fascicle) shortening velocities. This means that cadence is a possible confounding factor that was not accounted for in the current study. Nonetheless, we feel that the ecological validity benefits of this methodological approach offer more real-world insight into MTU interaction during cycling, albeit with the caveat that cadence, and in-turn MTU velocities, were not strictly controlled. The MTU model used in the current study is a two-dimensional model which does not account for the three-dimensional nature of muscle action^42^, meaning there is a risk of perspective error in *L*_*fasc*_ data. Additionally, this method does not consider the role of parallel-elastic element or other structural materials of the MTU (e.g., Titin). However, this method currently represents the best available approach for estimating MTU mechanical behavior during human movement. There is also an argument that ultrasound lacks the sensitivity to detect small changes that might exist between groups or conditions in a relatively constrained movement like cycling, perhaps because of the relatively low sampling frequency in comparison to other biomechanical measurement systems. Nonetheless, the current study’s sampling frequency was close to, or higher than, similar previous studies investigating MTU function during cycling^28,33^. This method of fascicle tracking can also be subject to tracking errors beyond those discussed above, induced either by the operator providing the algorithm with inaccurate initial conditions, or by the semi-automated tracking process itself. Given the frequency of the movement, these errors might be non-negligible and could be different between intensities (although this would be unlikely given a similar cadence). For this reason, we chose to only present *L*_*fasc*_ change data (relative to BDC), which is also more reliable than absolute data^34^. It should be noted that presenting length change data instead of absolute lengths could somewhat dampen the biological interpretation of the findings presented here (e.g., fascicle operating length with respect to the muscle’s length-tension properties), the importance of test-retest reliability when comparing between conditions should not be understated. For more information, the reader is directed to the Methods (Muscle-tendon function during cycling).

Overall, this study showed that the GM MTU underwent greater stretch and recoil during cycling at higher exercise intensities, but concomitant intensity-based changes in *L*_*fasc*_ or pennation angle were not found. Based on the length change data, GM fascicles stretched passively and to a lesser extent than the whole MTU, while the MTU also continued to stretch even after the fascicle began shortening. This MTU interaction implies some elastic energy storage, which is important for efficient endurance performance and aligns with established concepts of MTU interaction in other human movements. However, there were no significant differences detected in GM MTU neuromechanical behavior during cycling between groups using the current study design, even though TRI displayed higher AT thickness. Future research should consider exploring group differences in neuromechanical behavior at relative exercise intensities, and even under more mechanically constrained conditions (e.g., controlled cadences) where differences might be evident.

## Methods

### Participants

A convenience sample of 29 healthy men participated in this study, including 10 trained road cyclists (“CYC”; age: 39 ± 16 years; stature: 1.80 ± 0.09 m; body mass: 76.1 ± 9.3 kg), nine trained triathletes (“TRI”; 38 ± 11 years; 1.85 ± 0.09 m; 81.1 ± 8.6 kg), and 10 recreationally active controls (“CON”; 25 ± 4 years; 1.78 ± 0.03 m; 82.4 ± 9.1 kg). To be included in CYC or TRI, participants had to have a minimum of one year’s experience competing in their respective events. Four CYC self-reported to have 3–6 years of competitive experience, with a further two reporting 6–9 years and four reporting 10 years or more. Two participants from TRI reported having 1–3 years of competitive experience, with four reporting 3–6 years, one reporting 6–9 years, and two reporting 10 years or not more. At the time of testing, CYC self-reported a cycling and running volume of 262 ± 117 and 0 ± 1 km/week, respectively. TRI self-reported volumes of 122 ± 87 and 28 ± 23 km/week, respectively. CON self-reported volumes of 32 ± 34 and 15 ± 8 km/week, respectively. A one-way analysis of variances (ANOVA) (*F*_*2,26*_ = 17.95, *p* < 0.001, *η*_*p*_^*2*^ = 0.580 [large]) showed that CYC had higher cycling volume than CON (*p* < 0.001) and TRI (*p* = 0.005), whilst TRI and CON were not significantly different (*p* = 0.096). A one-way ANOVA (*F*_*2,26*_ = 9.53, *p* < 0.001, *η*_*p*_^*2*^ = 0.423 [large]) also showed that TRI had a higher running volume than CYC (*p* < 0.001) but not CON (*p* = 0.146), although CON and CYC were similar (*p* = 0.080). Participants provided written informed consent and were confirmed free of any musculoskeletal injury or disorder for at least six months before data collection. This research was approved by the Leeds Beckett University Local Research Ethics Committee and was conducted in accordance with the Declaration of Helsinki^43^.

### Data collection and processing

#### Muscle-tendon function during cycling

Cycling exercise testing was carried out on a customized Wattbike Pro cycle ergometer (Wattbike Ltd., Nottingham, United Kingdom). Ergometer customizations allowed us to replicate each participant’s habitual road cycling position, and are described in more detail elsewhere^44^. In brief, each participant’s saddle and pedals were transferred to the ergometer, and their cycling positional setup was replicated. If the participants did not have a specific road-racing bicycle (e.g., those in CON), the ergometer was fitted with a standard saddle, a crank length of 172.5 mm, and pedals with toe-straps, whilst ergometer geometry was adjusted to create a knee flexion angle of 25° at bottom-dead-center^45,46^, with the handlebar position in a comfortable but realistic road cycling position.

After a low-intensity 10-min warm-up (power output ~ 100 W)^47,48^, participants were required to cycle at a self-selected cadence for 5 min at four standardized intensities: 150, 200, 250, and 300 W, in a randomized order. Although cadence was self-selected for each participant, they were asked to keep that cadence consistent between conditions. This approach was taken to maximize ecological validity of testing whilst maintaining comparability between conditions, given changes in cadence would likely alter kinematic and neuromechanical parameters substantially. Constraining cadence (e.g., using isokinetic ergometry) could require participants to cycle at unfamiliar or suboptimal cadences, and would likely alter the within-revolution force-angle profiles, thus further altering MTU neuromechanical behavior. Between conditions, 5 min of active recovery was permitted to minimize fatigue accumulation. During the final 1 min at each intensity, pedal kinetics, joint kinematics, muscle activity, and muscle-tendon mechanical behavior was measured in the right leg and averaged over 20 consecutive pedal revolutions. Average cadence was also calculated from these revolutions (the inverse of the time required to complete the 20 revolutions, converted to revolutions per minute). Individual pedal cycles (from bottom-dead-center [BDC] to BDC) were determined using an accelerometer located within an instrumented pedal sensor sampling at 500 Hz (Powerforce; Radlabor, Freiburg, Germany). A duration of 5 min, with the final 1 min for data collection, was selected to allow participants to become familiarized and stabilized at the target intensity whilst minimizing the risk of inducing fatigue. If a participant showed signs of fatigue (e.g., a drop below the target intensity, a reduction in cadence, etc.), then they were removed from subsequent analyses.

**Fig. 5:**
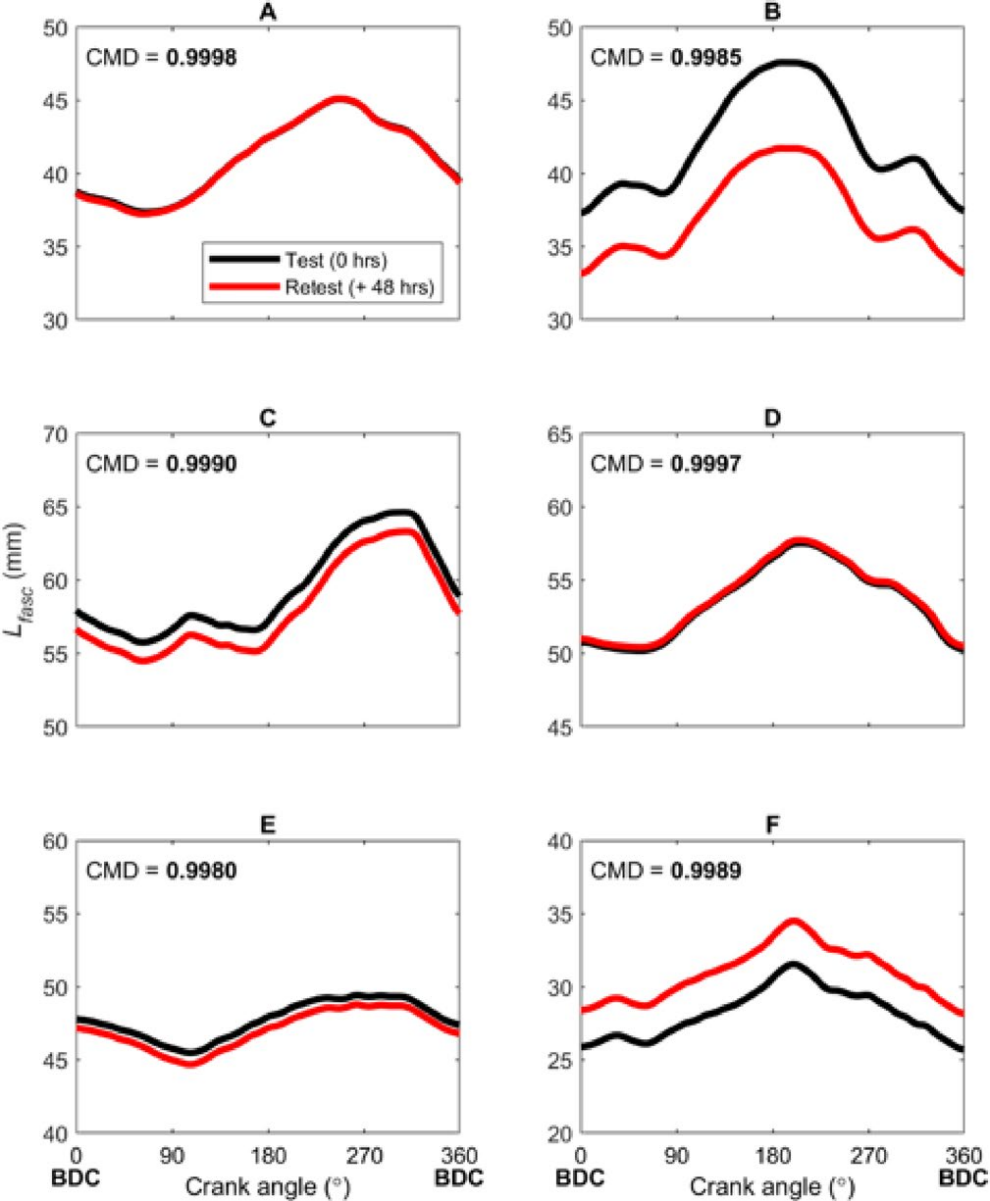
Test-retest reliability of fascicle length (*L*_*fasc*_) as a function of crank angle (BDC = bottom-dead-center). Each subplot (A-F) represents a single participant. CMD = coefficient of multiple determination, which is an indicator of waveform similarity, regardless of signal amplitude (where 0 and 1 represent perfect dissimilar and similar waveforms, respectively). All CMD values are > 0.99, indicating near-perfect waveform similarity despite random shifts in signal amplitude (i.e., absolute *L*_*fasc*_) between participants.

Sagittal-plane joint angles were measured with a four-camera motion capture system (Oqus 7+; Qualisys AB, Göteborg, Sweden) sampling at 250 Hz. Retroreflective markers were placed on the greater trochanter, lateral femoral epicondyle, lateral malleolus, calcaneus, and head of the fifth metatarsal whilst the participant was adopting a cycling-specific position. Marker positional data were filtered using a recursive second-order (zero phase-lag), low-pass digital Butterworth filter with a mean optimal cut-off frequency of 16.1 Hz, determined using residual analysis^49^. Joint positional data were then used to calculate joint angles throughout the pedal cycle. Ankle and knee angles were used to estimate GM MTU lengths using regression Eq.^20^ and individual participant shank length (distance from lateral femoral epicondyle to lateral malleolus). B-mode ultrasound videos of GM were obtained with an ultrasound scanner with 60-mm, 128-element linear array probe (LV7.5/60/128Z-2, 5.0–8.0 MHz; EchoBlaster 128 CEXT-1Z; Telemed UAB, Vilnius, Lithuania) recording at 60 Hz. The ultrasound probe was placed on the muscle belly of the right GM in line with the direction of muscle fibers and was fixed to the skin using a custom-made polystyrene casing and elasticated bandages. Specifically, three elasticated bandages were wrapped around the polystyrene casing which housed the probe, ensuring minimal probe translation and rotation during data collection, whilst a fourth bandage fixed the cable to a more distal point on the leg (above the ankle joint). This negated any cable tension around the probe to further minimize the risk of probe movement. In vivo recordings of *L*_*fasc*_ and pennation angle were tracked using a semi-automated tracking algorithm (UltraTrack, version 4.2)^34,50,51^. To account for temporal drift in the signal, key frame corrections were applied each time the right pedal reached BDC. All MTU data were presented as length change values, relative to BDC. The reason for this is that length change data are more reliably obtained from B-mode ultrasound videos than absolute *L*_*fasc*_ values because of human error induced when initially labeling the recording.^34^ To clarify this, we selected a random subset of ultrasound videos (*n* = 6 from the 200 W condition) and processed them on two occasions, separated by 48 h, by the same experienced researcher. Intraclass correlation coefficient (two-way mixed effects, single rater, absolute agreement) point estimate was 0.972 for average fascicle operating length (i.e., a value obtained from absolute *L*_*fasc*_ data), with a standard error of measurement of 2.46 mm, and 0.921 for fascicle operating range (the same whether obtained from absolute *L*_*fasc*_ or length change), but with a standard error of measurement of 0.76 mm. Figure 5 also depicts the waveform similarity of *L*_*fasc*_ as a function of crank angle, visualizing high waveform similarity between these two analyzed datasets, despite random shifts in signal amplitude (i.e., absolute *L*_*fasc*_). Therefore, the recommendations to present length change data, as opposed to absolute length data^34^, are supported.

Muscle activity data were collected for GM, GL, SOL, tibialis anterior (TA), VL using bipolar surface electromyography (Trigno wireless; Delsys Inc., Natick, MA) recording at 2,000 Hz. Each participant’s skin was prepared and electrodes were placed on the muscle belly and in line with the direction of fibers^52^. Raw electromyographic signals were processed similarly to Hug and Dorel^53^, where all data were normalized to the maximum activity within each trial to determine activity onset and offset (threshold set at 0.2). To compare amplitude between cycling intensities, signals were normalized to the maximum activity detected during the 300 W condition. This meant that activity magnitudes could be compared between intensities without affecting within-condition onset and offset calculations. All systems described above (kinematics, ultrasound, and electromyography) were synchronized by sending a 5-V Transistor-Transistor-Logic signal from the ultrasound unit to trigger other systems.

## Muscle and tendon morphology

Static two-dimensional images of various muscle and tendon sites on the right leg were obtained using B-mode ultrasound (Acuson P300; Siemens Healthineers AG, Erlangen, Germany). Using a 50-mm linear-array probe (5–12 MHz), longitudinal-plane scans of GM, GL, and SOL were obtained in the anatomical standing position^54^ by the same operator. GM and GL muscle bellies were estimated to be 30% of the distance from the lateral femoral epicondyle to the lateral malleolus, with the final location being adjusted to account for anatomical variation and anatomical obstructions (e.g., blood vessels). SOL muscle belly locations were estimated in the same way but using 50% of the distance between lateral femoral epicondyle and lateral malleolus. Once muscle bellies were located, the ultrasound probe was placed in the longitudinal plane, on the mid-sagittal axis of the muscle belly, and in the direction of the muscle fibres^55^. Images were analyzed manually using Fiji (ImageJ 1.52i, 64-bit; National Institutes of Health, Bethesda, MA) for muscle thickness (distance between superficial and deep aponeuroses), pennation angle (angle between line of fascicle and deep aponeurosis), and *L*_*fasc*_ (straight-line distance between a fascicle’s insertion sites at the deep and superficial aponeuroses). When a full fascicle was not visible in the field-of-view, a manual linear extrapolation method^56–58^ was used and cross-checked with an alternative estimation method^59^, which extended the superficial aponeurosis beyond the field-of-view and projected the fascicle to the insertion point. Using a 40-mm linear-array probe (12–18 MHz) from the same unit, longitudinal-plane scans of the AT were obtained 40 mm proximal to the calcaneal insertion. Using Fiji, tendon thickness was measured as the distance between deep and superficial edges of the tendon (including peritenon)^60,61^. For each site, three images were obtained and analyzed independently, and the mean value across images for each variable was taken forward for analysis. High test-retest reliability of manually analyzing images in endurance-trained populations has previously been shown^60^, but to ensure reliability, a random subset of images from one muscle (GM) and AT were analyzed on two occasions, separated by 48 h, by the same experienced researcher. Intraclass correlation coefficient (two-way mixed effects, single rater, absolute agreement) point estimates were 0.999, 0.715, and 0.920 for GM muscle thickness, pennation angle, and *L*_*fasc*_, respectively (standard error of measurement = < 0.1 mm, 1.3°, and 0.9 mm), and 0.952 for AT thickness (standard error of measurement = < 0.1 mm).

### Statistical analysis

Statistical analyses were carried out in SPSS (version 26; IBM, Armonk, NY). Following the calculation of descriptive statistics, the data were screened for normality, homogeneity of variances, and sphericity. A series of one-way and two-way analyses of variances (ANOVA) were used to detect differences between groups and conditions, as well as group × condition interactions where relevant. In the event of significant main effects or interactions, pairwise Bonferroni-corrected comparisons were employed. Effect sizes of main effects and interactions were estimated with partial eta-squared (*η*_*p*_^*2*^), which were interpreted with the following benchmarks: <0.010 = negligible, 0.010–0.059 = small, 0.060–0.139 = medium, and ≥ 0.140 = large^62^. In addition to discrete comparisons, statistical parametric mapping (SPM) two-way ANOVA with repeated measures tests^63^ were carried out using ‘spm1d’ (version M.0.4.7) in MATLAB (version 2020b; MathWorks Inc., Natick, MA) to compare normalized time-series data between groups and conditions, as well as group × condition interactions. The addition of SPM analysis to conventional discrete comparisons enabled a greater appreciation for where in the pedal cycle differences exist. Post-hoc testing was not conducted for SPM analyses, as no validated tests are available at present. Therefore, any individual group- or intensity-based differences were interpreted observationally. For all tests, the significance level was set at *p* < 0.05.

## Supplementary Information

Below is the link to the electronic supplementary material.

Supplementary Material 1

## Data Availability

The datasets generated during and/or analysed during the current study are not publicly available due to the involvement of human participants but are available from the corresponding author on reasonable request.
