# Supplementary material for: Gastrocnemius medialis neuromechanics during cycling at various exercise intensities
Source: Sci Rep. 2026 May 2;16:20309. doi: 10.1038/s41598-026-51412-2 (PMC13324757; doi:10.1038/s41598-026-51412-2)

**Supplemental Fig. S1.** Statistical Parametric Mapping (SPM) results for knee and ankle joint angle profiles.


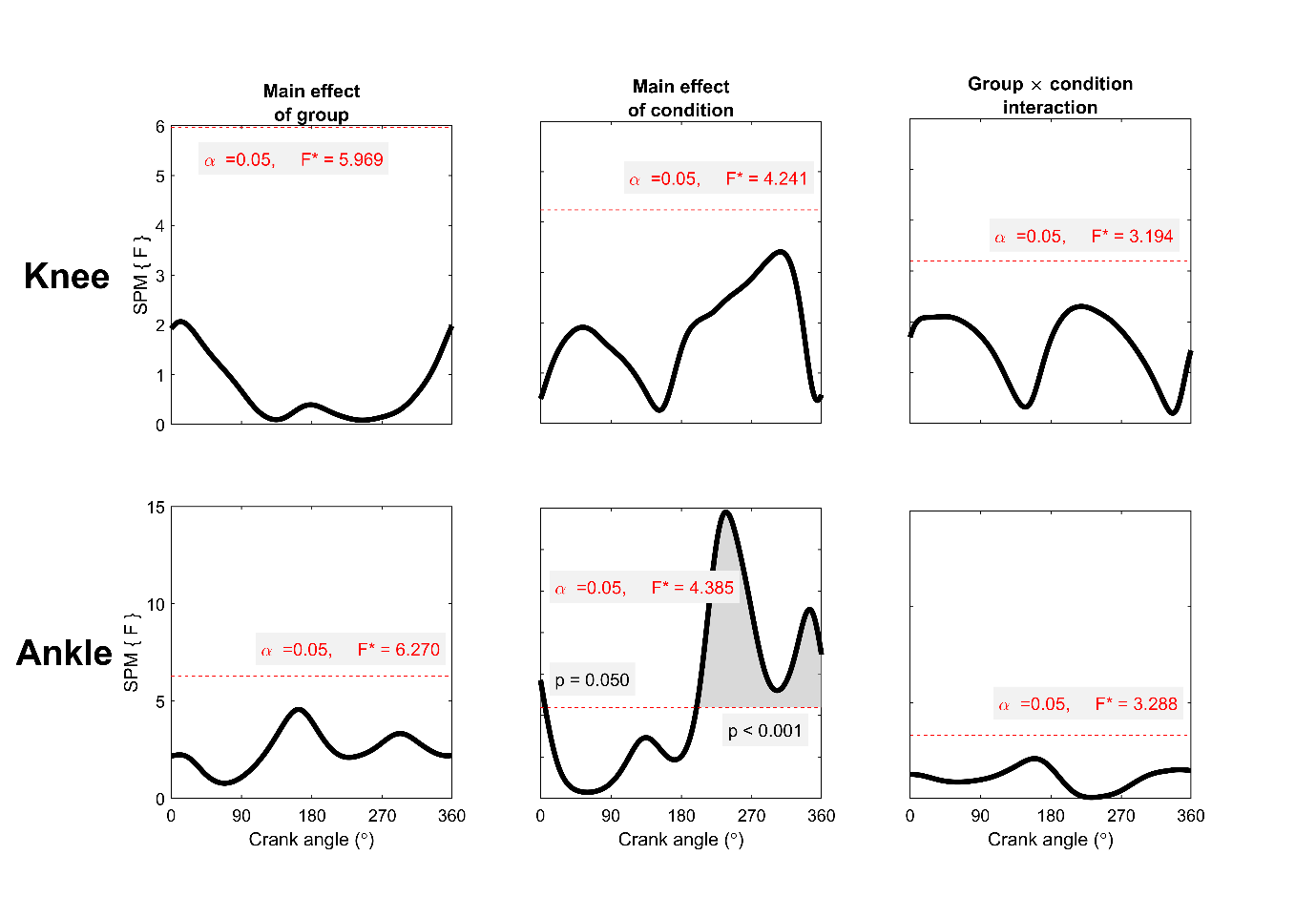


**Supplemental Fig. S2.** Statistical Parametric Mapping (SPM) results for muscle activity profiles.


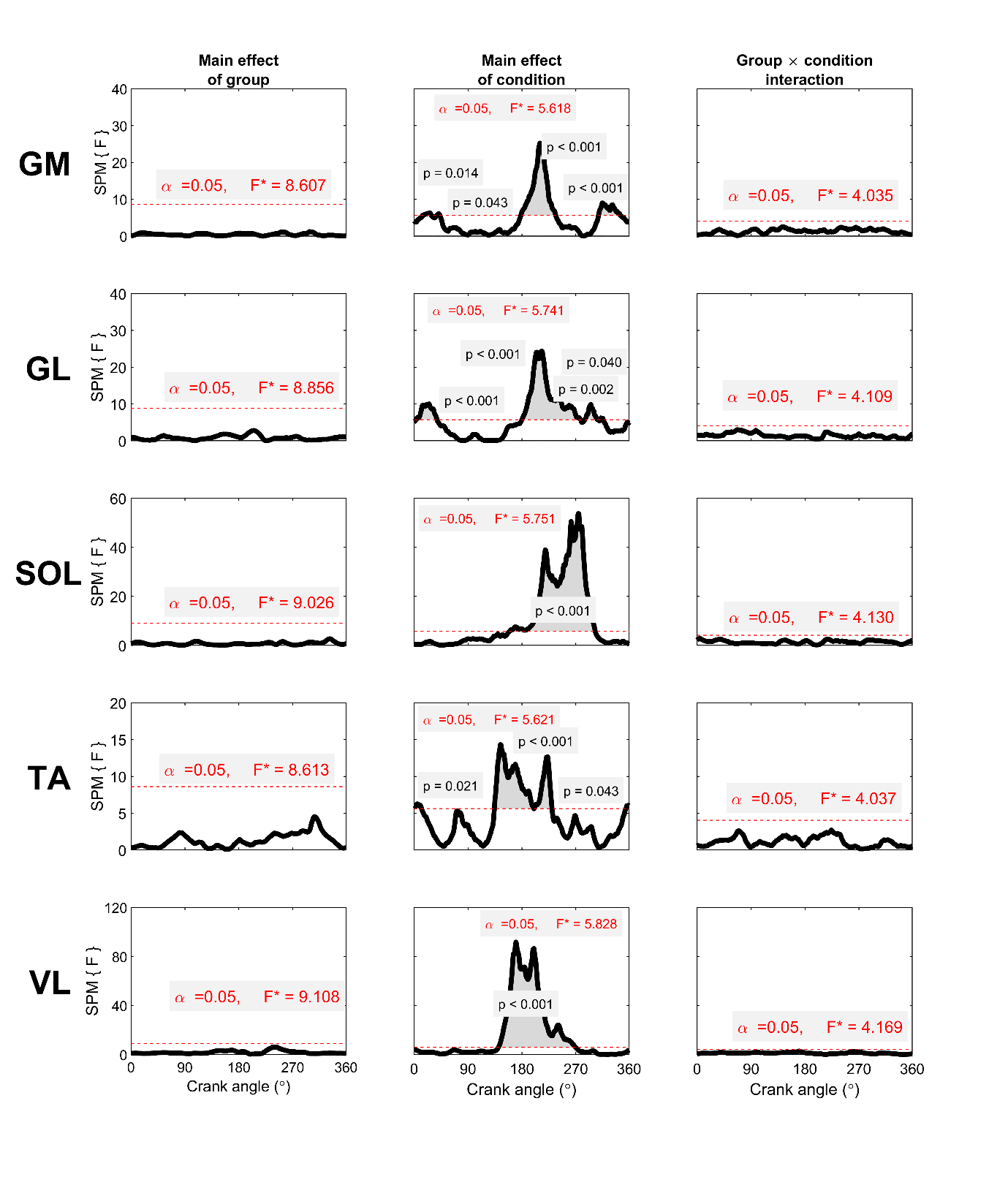


**Supplemental Fig. S3.** Statistical Parametric Mapping (SPM) results for muscle-tendon unit mechanical characteristics.


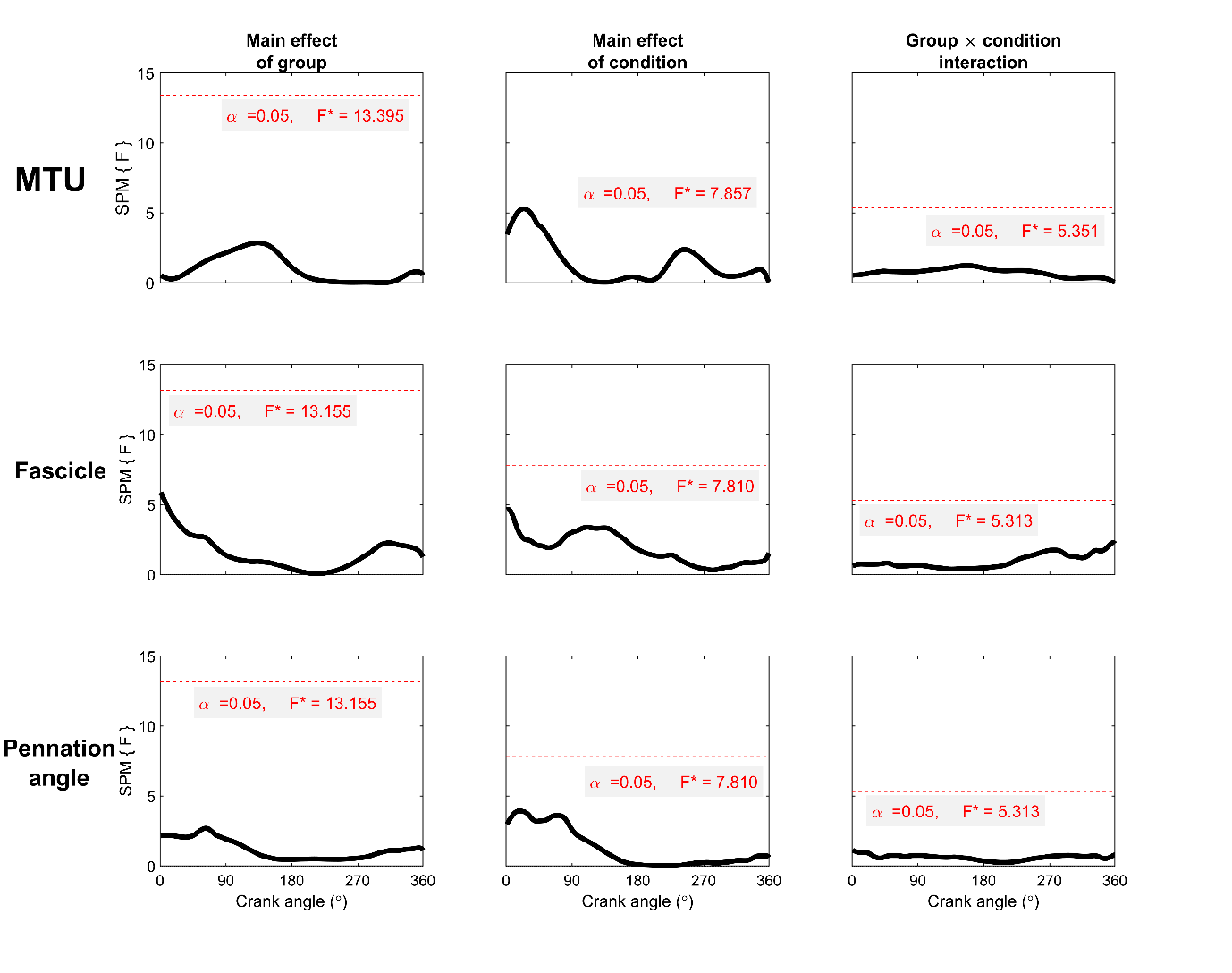

Supplement: Supplementary file 1 — Supplementary Material 1 [file 41598_2026_51412_MOESM1_ESM.docx]
